# Opening a window to the acutely injured brain: Simultaneous retinal and cerebral vascular monitoring in rats

**DOI:** 10.3389/fnmol.2023.1116841

**Published:** 2023-03-24

**Authors:** Laura Warner, Annika Bach-Hagemann, Tobias P. Schmidt, Sarah Pinkernell, Gerrit A. Schubert, Hans Clusmann, Walid Albanna, Ute Lindauer, Catharina Conzen-Dilger

**Affiliations:** ^1^Translational Neurosurgery and Neurobiology, Department of Neurosurgery, Medical Faculty, RWTH Aachen University, Aachen, Germany; ^2^Fraunhofer Institute for Toxicology and Experimental Medicine, Department of Preclinical Pharmacology and Toxicology, Hannover, Germany; ^3^Department of Neurosurgery, Medical Faculty, RWTH Aachen University, Aachen, Germany; ^4^Department of Neurosurgery, Kantonsspital Aarau, Aarau, Switzerland; ^5^Institute for Neurophysiology, University of Cologne, Cologne, Germany

**Keywords:** retinal vessel analysis, non-invasive, blood flow assessment, rat, neurovascular coupling

## Abstract

Many recent research projects have described typical chronic changes in the retinal vasculature for diverse neurovascular and neurodegenerative disorders such as stroke or Alzheimer's disease. Unlike cerebral vasculature, retinal blood vessels can be assessed non-invasively by retinal vessel analysis. To date, there is only a little information about potential simultaneous reactions of retinal and cerebral vessels in acute neurovascular diseases. The field of applications of retinal assessment could significantly be widened if more information about potential correlations between those two vascular beds and the feasibility of non-invasive retinal vessel analysis in acute neurovascular disease were available. Here, we present our protocol for the simultaneous assessment of retinal and cerebral vessels in an acute setting in anesthetized rats using a non-invasive retinal vessel analyzer and a superficial tissue imaging system for laser speckle contrast analysis *via* a closed bone window. We describe the experimental set-up in detail, outline the pitfalls of repeated retinal vessel analyses in an experimental set-up of several hours, and address issues that arise from the simultaneous use of two different assessment tools. Finally, we demonstrate the robustness and variability of the reactivity of retinal vessels to hypercapnia at baseline as well as their reproducibility over time using two anesthetic protocols common for neurovascular research. In summary, the procedures described in this protocol allow us to directly compare retinal and cerebral vascular beds and help to substantiate the role of the retina as a “window to the brain.”

## 1. Introduction

The dysfunction of the neurovascular unit affects neurovascular, neurodegenerative, and neurooncological diseases. However, the limited accessibility of cerebral vessels complicates functional diagnostics for both physicians and fundamental researchers. In the clinical setup, the different imaging techniques for cerebral circulation are often time-consuming and expensive. As patients need to be transported to the diagnostic unit, they have a higher risk of complications and require extensive manpower (Donnelly et al., 2016). Furthermore, the results of the performed diagnostics just offer a snapshot of the current situation of cerebral vessels that can quickly change. Although bedside monitoring of the brain function and cerebral vessels [e.g., microdialysis (Hutchinson et al., 2015) and ptiO2 (Maloney-Wilensky et al., 2009)] exists, it is invasive for most patients as they require surgery with potential adverse effects such as bleeding or wound infection. The invasive monitoring can extend to a longer measuring period of several days to weeks; however, information is often limited to a small local area of brain tissue of several centimeters. In addition, the obtained data are processed *via* complex algorithms, further limiting the use of bedside monitoring for research purposes (Nordström et al., 2017). In the experimental set-up, cerebral vessel monitoring and especially functional testing are often associated with large surgical procedures that need highly trained personnel, require longer anesthetics with the risk of further complications such as repetitive anesthesia for long-term observations, or call for highly demanding training of animals when used in awake settings (Hoover et al., 2021; Sciortino et al., 2021). Therefore, these procedures may be even restricted to acute settings. For the clinical and the experimental set-ups, simplifying this monitoring would allow gaining more information about the cerebral vessel function and the neurovascular unit in particular, which could directly influence the timepoint of treatment decisions.

The retina is an embryologically original part of the central nervous system and, in terms of vascular functions and morphology, shares important features with the brain (Cabrera DeBuc et al., 2017): the retinal vasculature also hosts the mechanisms of neurovascular coupling, autoregulation, and vascular reactivity to CO_2_ (Kur et al., 2012). Easy access to the retina is a major advantage for diagnostic assessment. Techniques, such as retinal vessel analysis, provide direct insights into retinal microcirculation and vasculature in a non-invasive and bedside fashion. Recently, several studies have examined the retina as a proxy for the brain (“window to the brain”), reporting a link between retinal pathologies and neurodegenerative or cerebrovascular diseases (London et al., 2013). Variations in retinal arteriolar microvascular functions and microcirculatory properties of the brain (measured by MRI intravoxel incoherent motion) are linked, potentially enabling early detection of brain microvascular dysfunction (van Dinther et al., 2022). Recently, our group observed the first clinical evidence of retinal vasculature changes after aneurysmal subarachnoid hemorrhage (SAH) (Albanna et al., 2016, 2021; Conzen et al., 2018). Finally, we have presented another link between cerebral and retinal vasculatures in an acute neurovascular experimental set-up: The vascular reactivity to hypercapnia was simultaneously impaired in both vascular beds, retinal and cerebral, in the acute phase after experimental SAH in rats (Warner et al., 2022). Our results showed that minutes after SAH, retinal vessels participated in changes of the cerebral vessels, suggesting that the retinal vessels were directly affected by the same impact and therefore may serve as easily accessible proxies for acute cerebral vascular changes.

This article presents our step-by-step protocol for simultaneous retinal and cerebral vessel assessment by means of the Imedos-Retinal Vessel Analyzer (RVA) and a custom-made laser speckle system. In an experiment lasting several hours, we outline pitfalls concerning repeated retinal vessel analyses and address issues arising from the simultaneous use of two different assessment systems. We give application examples of vascular reactivity to a hypercapnia challenge in both vascular beds, cerebral and retinal, characterizing three types of retinal vessel reactivity. Finally, we present our experience with two common anesthetic protocols for neurovascular research.

## 2. Materials and equipment

All materials used in the experiments are listed in Table 1.

**Table 1:** Inventory of all the materials used.

**Animal housing**	
**Material**	**Manufacturer**
Cagetype 2000P	Tecniplast, Hohenpreisenberg, Germany
Wood litter ¾ S	Rettenmeier, Ramstein-Miesenbach, Germany
Food (V1534-300)	Ssniff, Soest, Germany
**Medication**	
**Substance**	**Manufacturer**
Isoflurane Forene^®^	Abbvie Ludwigshafen, Germany
Fentanyl 0.5 mg/10 ml	Rotexmedica, Trittau, Germany
Ropivacain 2 mg/ml, Solution for injection	Fresenius Kabi Deutschland GmbH, Bad Homburg, Germany
Sodium chloride 0.9%, Solution for injection	Braun Melsungen AG, Melsungen, Germany
Ketamin 100 mg/mL	Medistar Arzneimittelvertrieb GmbH, Ascheberg, Germany
Xylazin 20 mg/mL	Wirtschaftsgenossenschaft deutscher Tierärzte eG, Garbsen, Germany
Mydraiticum	Pharma Stulln GmbH, Stulln, Germany
Eye ointment: Corneregel	Bausch + Lomb GmbH, Berlin, Germany
Pancuronium 4 mg/ 2 mL	Inresa Arzneimittel GmbH, Freiburg im Breisgau, Germany
Vecuronium 10 mg	Inresa Arzneimittel GmbH, Freiburg im Breisgau, Germany
**Surgery**	
**Material**	**Manufacturer**
Isofluran Vetmed Vapor	Drägerwerk AG, Lübeck, Germany
Rodent Ventilator (7025)	UGO BASILE Gemonio, Italy
Temperature Control Unit HB 101/2; plate (76-0386); Temperature probe for mouse/ rats (76-0067)	Harvard Apparatus Ltd., Kent, GB
Stereotact	WPI, Friedberg, Germany
Pulse oximeter	Harvard Apparatus Ltd., Kent, GB
Blood pressure monitor BLPR2 and SYS-BP1	WPI, Friedberg, Germany
ICP- monitor BLR2 and SYS-BP1	WPI, Friedberg, Germany
Programmable single syringe pump NE-300 TM	New Era Pump Systems, Inc., Farmingdale, USA
Laser speckle contrast analysis system: Superficial Tissue Imaging System, STIS	Biomedical Optics Laboratory, RheinAhrCampus Remagen, Germany
Surgery Microscope Olympus SZX7	Olympus, Tokyo, Japan
Surgery light: Olympus KL 1500 LED	Olympus, Tokyo, Japan
Microdriller Micromot 50/E, diamond mounted point (28212)	PROXXON, Wecker, Luxembourg
Blood gas analyzer combi line	Hadler and Braun GmbH and Co. KG, Eschweiler, Germany
Surgery instruments	Fine Science Tools, Heidelberg, Germany
Syringes, needles	Braun, Melsungen, Germany
Polythene Tubing for intubation *via* tracheotomy (2.42 mm OD, 1.67 mm ID)	Smiths Medical International Ltd., Kent, GB
Polythene Tubing for femoral artery and vein catheter (0.96 mm OD, 0.58 mm ID)	Smiths Medical International Ltd., Kent, GB
Polythene Tubing for cisterna magna / ICP catheter (0.61 mm OD, 0.28 mm ID)	Smiths Medical International Ltd., Kent, GB
Hoffmanns's AQUACC Carboxylate Cement	Hoffmann Dental Manufaktur GmbH, Berlin, Germany
Arterial Blood Sampler Aspirator	Radiometer Medical ApS
Lens	Ocular Instruments, Bellevue, USA
Camera	Imedos Systems GmbH, Jena, Germany
Seraflex USP 0	Serag Wiessner, Naila, Germany
Seraflex USP 4-0	Serag Wiessner, Naila, Germany
Cotton buds	Paul Boettger GmbHand Co. KG, Bodenmais, Germany
Swaps: Zelletten, 4 × 5 cm	Lohmann and Rauscher GmbH and Co.KG, Neuwied, Germany
Parafilm	Witeg, Wertheim am Main, Germany
Bonewax	Ethicon Endo-Surgery, Cincinnati, Ohio, Vereinigte Staaten
Cover slip	Carl Roth GmbH + Co. KG, Karlsruhe, Germany
Glue	Uhu GmBH and Co. KG, Bühl/ Baden, Germany
Micromanipulator C-2	Narishige Scientific Instrument Lab., Tokyo, Japan
Hydraulic lifting jack (ConStands)	Motea GmbH, Wiehl, Germany
**Software**	
**Software**	**Manufacturer**
Spike2 Version 8.02e	Cambridge Electronic Design, Cambridge, GB
GraphPadPrism 7.04	GraphPad San Diego, Kalifornien, USA
Excel 2016	Microsoft Cooperation, Washington, USA
EndNote X7.8	Thomson Reuters, New York City, USA
MATLAB 7.3.0.267/R2006b	The MathWorks, USA
MATLAB Skript	Steimers et al., Biomedical Optics Laboratory, RheinAhrCampus Remagen, Deutschland
Imedos RVA	Imedos Systems GmbH, Jena, Deutschland

## 3. Methods

This study aimed to present a procedure for the simultaneous assessment of retinal and cerebral vessels in the rat using a non-invasive retinal vessel analyzer and a laser speckle system *via* a closed brain window. With this method, the effects of acute neurovascular events such as hemorrhage, stroke, or trauma can be monitored live. Our procedure allows us to directly compare both vascular beds and helps to substantiate the role of the retina as a “window to the brain.” We describe the surgical preparation in detail and specify the complex experimental set-up for the assessment of cerebral blood flow (CBF) by the laser speckle analysis and of retinal vascular diameters by means of the Imedos-RVA for small animals. In addition, two anesthetic protocols and the narcotics' effects on the cerebral and retinal vessels are described. The demonstrative examples used in this article are taken from the analysis performed in the context of our previous, already published study (Warner et al., 2022). Statistical analyses between and within anesthetic groups can be found in that study.

### 3.1. Experimental animals

All experiments were performed in accordance with the German Animal Welfare Act and the EU Directive 2010/63 under permit number 84-02.04.2015.A412 (LANUV, Recklinghausen, Germany).

For this protocol, male Wistar rats (Janvier Labs, Le Genest-Saint-Isle, France) with a weight of ~300 g were housed in groups of two or three at the Institute for Laboratory Animal Science of RWTH Aachen University (quality management certified according to ISO9001:2015) in type 2000P cages, with food (V1534-300, Sniff, Soest, German) and water *ad libitum*. All rats were kept on a 12-h light–dark cycle (07:00–19:00), a temperature of 22 ± 2 degrees, and a humidity of 55 ± 5%. They were submitted to an adaptation period of at least 7 days before being used in experiments. Health monitoring was performed according to the Federation of European Laboratory Animal Science Associations (FELASA) recommendations (Mähler et al., 2014).

### 3.2. Medication and solutions

As corneal desiccation can significantly affect imaging quality, it is essential to repeatedly moisturize the rats' eye with a transparent eye ointment and cover it with a coated swab (conventional swab wrapped with parafilm) right after anesthesia induction. To prevent undesired eye bulb rotation (see Supplementary Video 1) and to ensure a high quality of retinal vessel assessment over the measurement period, the use of a muscle relaxant is recommended. In this protocol, two drugs were used with comparable good results: pancuronium bromide (1.5 mg/kg every 2 h, administered intraperitoneally) or vecuronium (7.8 mg/kg/h, administered by continuous infusion) starting right before the preparation of the cranial window. In this case, additional monitoring parameters are required to regularly check the sufficient depth of anesthesia since testing of the pain reflexes is no longer possible in muscle-relaxed animals.

Retinal measurements require a fully dilated pupil. The use of muscarinic antagonists is recommended as alpha antagonists can influence the diameter of the retinal vessels and thus falsify the results (Garhofer et al., 2010). In this protocol, tropicamide was used as mydriatic (Pharma Stulln GmbH, Stulln, Germany) and applied first 15 min before baseline and then every 2 h.

#### 3.2.1. Anesthesia

**Figure 1:**
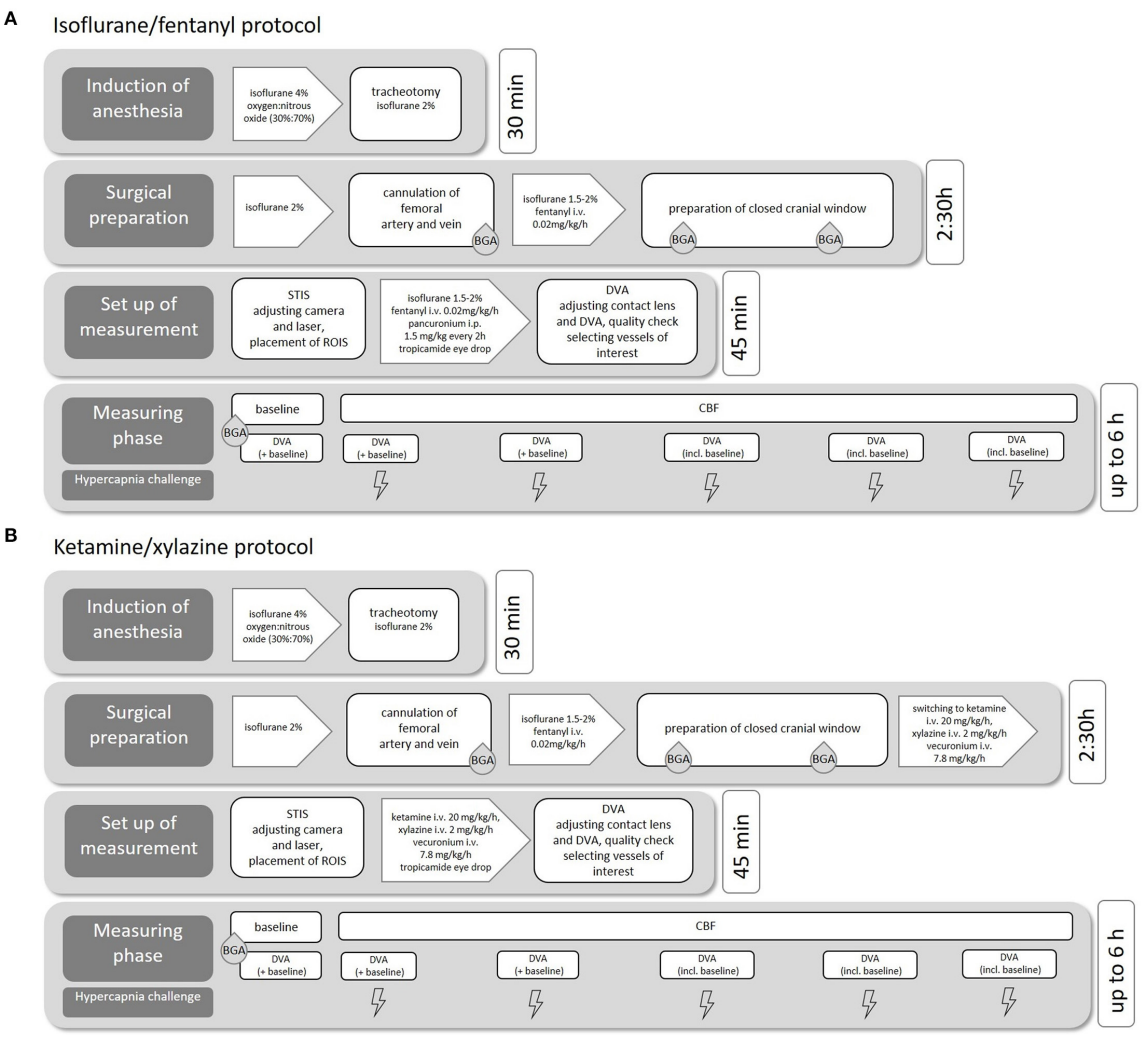
Experimental procedure. Flowchart of the individual surgical steps including medication and estimated duration for isoflurane/fentanyl anesthesia **(A)** and ketamine/xylazine anesthesia **(B)**.

We performed the experiments with two common anesthetics protocols (see Figure 1A) to evaluate the effects of the narcotic medication on cerebral and retinal vessel diameters. In the first protocol, we used isoflurane anesthesia combined with fentanyl for analgesia (isoflurane/fentanyl; Iso-Group), which is well-known and established in our research group. Notably, isoflurane is a very potent vasodilator, and other studies have shown its enormous vasodilatory effect on the retina. This effect must be considered in retinal vascular measurements (Muir and Duong, 2011).

We, therefore, studied in a second protocol of another group with a combination of ketamine and xylazine (ketamine/xylazine; K/X group, see Figure 1B). This protocol was adopted from a study that evaluated the effect of different anesthetic protocols on rat ocular blood flow (Moult et al., 2017). In both anesthesia regimes, ropivacaine (2 mg/ml) was injected subcutaneously for local analgesia before all skin incisions.

##### 3.2.1.1. Isoflurane fentanyl anesthesia

The induction of anesthesia was performed with isoflurane in a mixture of oxygen and nitrous oxide. The concentration of isoflurane was adjusted quickly and easily during the operation/measurement phase, depending on the rat's vital parameters and the results of typical reflex tests. After successful cannulation of the femoral vein, additional analgesia was provided by continuous infusion of fentanyl (0.02mg/kg/h).

##### 3.2.1.2. Ketamine/xylazine

The induction of anesthesia was performed with isoflurane and fentanyl as described earlier. After the implantation of the closed bone window, anesthesia was slowly switched from Iso to K/X (ketamine, K: 20 mg/kg/h, xylazine, X: 2 mg/kg/h) *via* continuous intravenous infusion: for this purpose, the fentanyl continuous infusion system was replaced by a syringe pump containing ketamine, xylazine, and vecuronium. As soon as a drop in blood pressure occurred (approximately after 5–10 min), the isoflurane flow was reduced to 0.5–1%. After another 10 min, the supply of isoflurane was stopped.

### 3.3. Protocol of the surgical procedure

**Figure 2:**
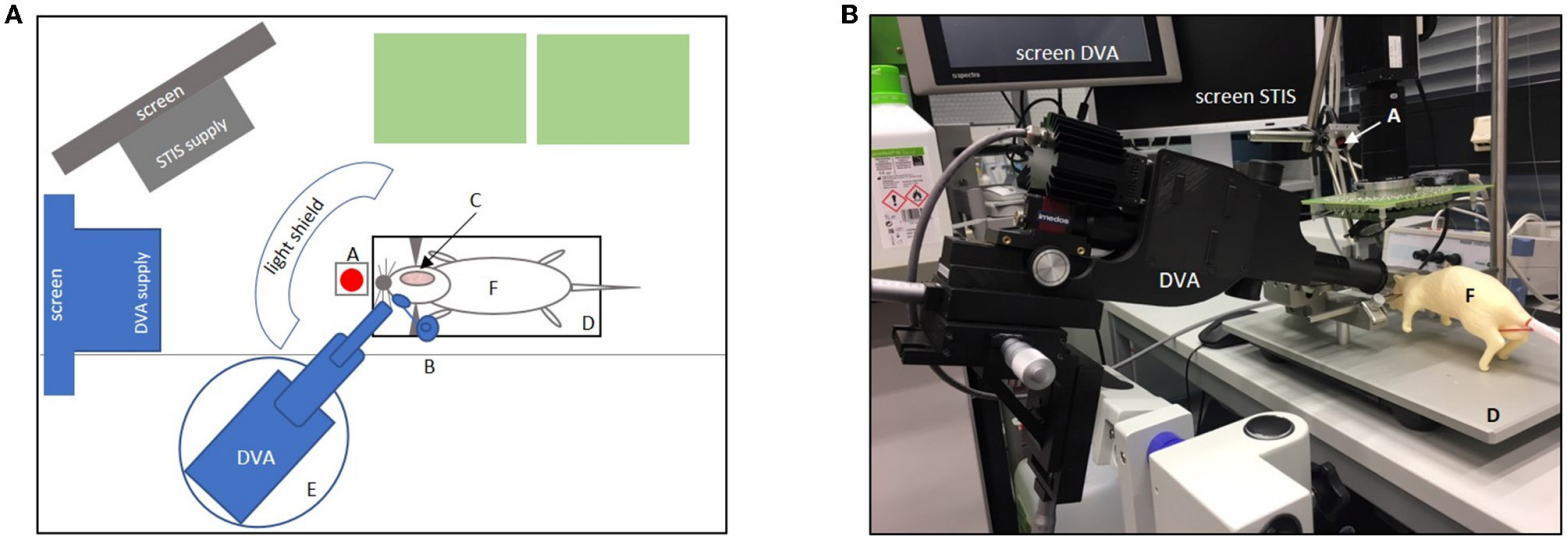
Experimental set-up. **(A)** Shows the set-up in the aerial view. Green boxes = life support supplies (e.g., respirator, Perfusor, and blood pressure monitor); gray boxes = computer screen with laser speckle system (STIS) supply; gray half-moon = light shield; blue boxes = dynamic vessel analyzer (DVA) and DVA supply including a screen for video analysis (A, laser diode for laser speckle contrast; B, micromanipulator with a contact lens placed in front of the right eye; C, cranial bone window; D, stereotactic frame with heating plate and animal in supine position; E, lifting jack for the adjustment of the DVA; F, dummy. **(B)** Original set-up with dummy showing the DVA with DVA supply screen and a second screen for laser speckle imaging (STIS). A, laser speckle system (STIS); D, stereotactic frame; F = dummy. Micromanipulator with contact lens and light shield not shown here.

**Figure 3:**
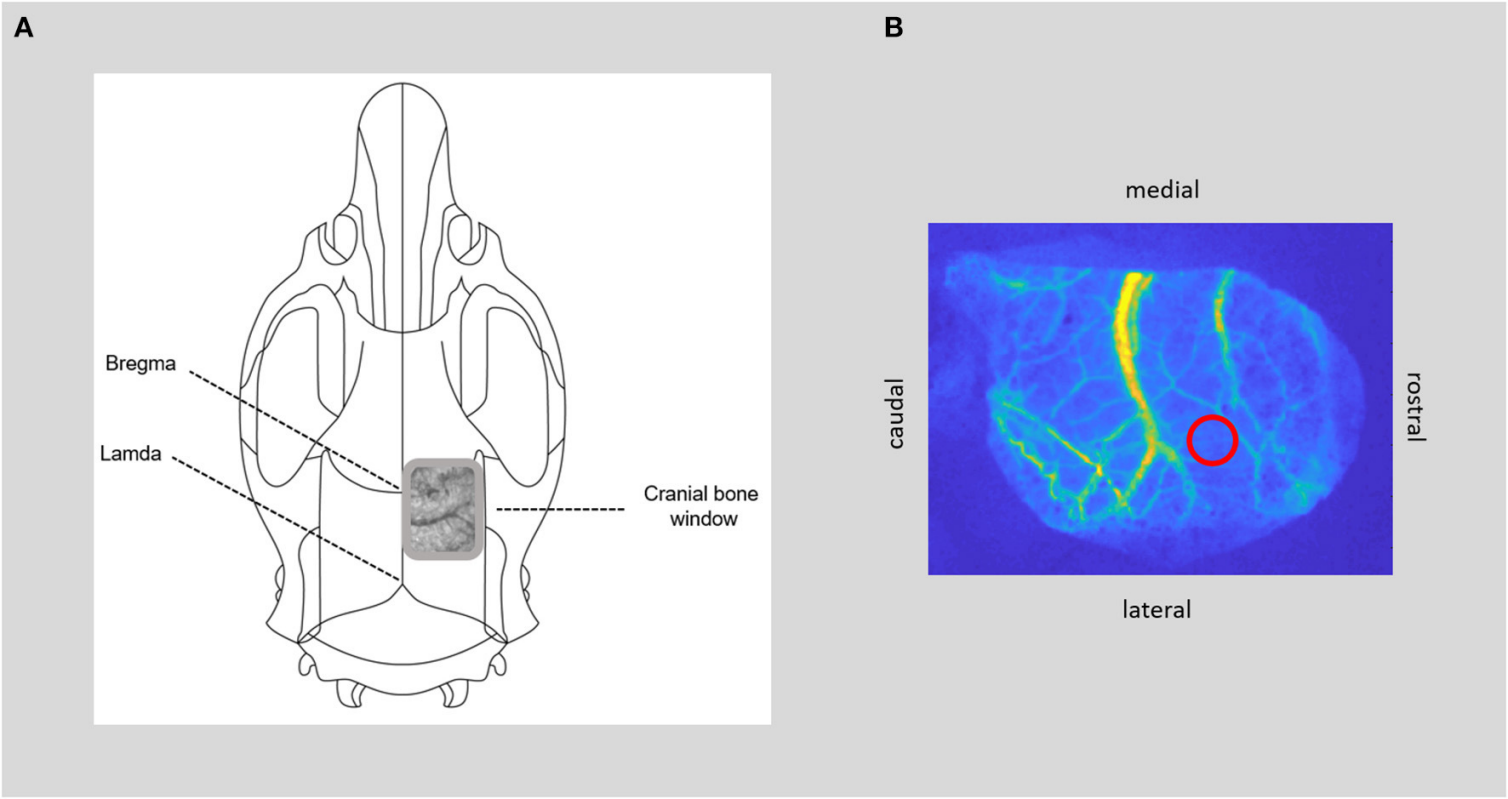
CBF measurement. **(A)** Positioning of the bone window over the somatosensory cortex, with thinned bone and visible cerebral vessels. **(B)** Image of cerebral blood flow calculated from raw laser speckle images with the region of interest within the microcirculation (red circle) for time course analysis of cerebral blood flow.

In the following, we present our protocol for the surgical procedures and the step-by-step set-ups of cerebral blood flow measurement and retinal vessel analyses (see Figures 2, 3 for an overview).

Approximate surgical time: 3 h and 30 min.

#### 3.3.1. Pre-operative preparation

- Prepare all required surgical instruments and consumable supplies including suture material and endotracheal tubes.
- Clean all surgical surfaces with 70% denatured ethanol.

#### 3.3.2. Surgical procedure

- Start every surgical procedure at the same time, preferably in the morning.
- Perform standard procedures of tracheotomy for artificial ventilation and cannulate the femoral artery and vein on one side to assess arterial blood pressure and blood gas analysis and to apply i.v. medication (systemic analgesia, anesthesia in K/X, and muscle relaxation).
- *Note: Routinely monitor respiration rate, body temperature, heart rate, systemic oxygen saturation, and systemic arterial pressure as well as arterial blood gases throughout surgery to maintain physiological cardiac and respiratory functions. The respiratory rate is set based on regular blood gas analyses every 60 min and adjusted if necessary. Before the application of muscle relaxant, routinely test reflexes to determine a sufficient depth of the anesthesia. After the application of the muscle relaxant, check the sufficient depth of anesthesia via control of heart rate and blood pressure*.
- For cranial window implantation, place the animal in a prone position in a stereotactic frame after injecting ropivacaine to the base of both ears for ear bar fixation.
- Prepare the closed cranial window (see Figure 3): After subcutaneous injection of local analgesia, make a small incision with scissors at the posterior edge of the occipital bone and remove the skin to expose the skull bone. Remove connective tissue from the skull. Detach the temporal muscle from the lateral ridge of the parietal bone and dissect the upper parts of the muscle to make room for window installation. *Note: At this step, it is very important to prevent bleeding from the surrounding tissue as continuous bleeding will interfere with the speckle contrast imaging later*.
- Determine the position of the cranial window over the somatosensory cortex with the center of the window ~8 mm caudal and 2.5 mm lateral to bregma and ~12 × 5 mm in size over the right hemisphere. Then, thin the skull bone with the drill under constant cooling with saline until the cerebral vessels are visible and the skull starts to become flexible. A very thin bone lamella should be preserved, still covering the brain. Regularly test the softness of the skull with forceps by gently pressing on the drilled area. The skull bone is thin enough to be easily depressed, while the bone lamella remains intact. The window is completed when the cerebral vessels are visible, and the bone is thinned out evenly in all areas.
- *Note: In our experiments, we performed this step under an operating microscope. It is very important to grind down the skull bone without too much pressure as any bleeding/trauma of the brain (e.g., subdural hematoma) will interfere with laser speckle contrast and may impair vascular reactivity later. Give special attention to the area of the coronal suture as bleeding will occur more frequently here. An effective way to stop bleeding from the skull is to drill without saline for a short period of time. We avoided the use of hemostatic products as vasoconstriction may potentially falsify the results of the cerebral blood flow measurement*.
- If there is no active bleeding anymore, place an ~2-mm-high wax wall around the window. Make sure that the skull bone and the area around the window are completely dry. Place a prepared wax wall around the window and smooth the inner and outer parts of the wax wall out to the edge of the window to ensure water tightness. *Note: The wax wall must be watertight; we recommend testing this before covering it with a cover slip*.
- Fill the window with saline and then cover it with a cover slip to ensure stable conditions for the speckle analysis throughout the measuring period.
- Proceed with the set-up of the required measuring tools.

### 3.4. Protocol of cerebral blood flow measurement

- Set-up of the laser speckle system (STIS, Biomedical Optics Laboratory, RheinAhrCampus, Remagen, Germany): the used CCD camera (AD-080GE, Jai A/S, Denmark), with 4.65 μm × 4.65 μm pixel size and 1,024 pixel × 768 pixel resolution, has a dynamic range of 12 bit and a final frame rate of 0.5 Hz (average of 30 frames). The moving average was set to 10, and an exposure time of 10,000 μs was used.
- Place the animal under the STIS while remaining in the stereotactic frame to constantly measure and record CBF free of moving artifacts (Conzen et al., 2019; Warner et al., 2022).
- *Note: Avoid artificial movements, e.g., for controlling the ventilation tubing or accidental bumping on the stereotactic frame as these interfere with the measurement*.
- Place the bone window centrally under the laser speckle system camera. Using the zoom and the sharpness settings, adjust the camera in a way that the cranial window fills the entire camera image. The optimal lighting conditions are set by the system.
- Align the laser with the cranial window to capture the entire area.
- Place regions of interest (ROIs) within the microcirculation of the somatosensory cortex. *Note: CBF from raw speckle images is subsequently evaluated offline as published before (Steimers et al., 2013; Conzen et al., 2019) Measurements of CBF are normalized to the baseline (5 min) recorded before each hypercapnic stimulation period*.

### 3.5. Protocol of retinal vessel diameter measurement

#### 3.5.1. Set-up

- Assemble the retinal camera (DVA) on a small vibration-damped lifting jack to allow 360 degrees of movement. Shield the set-up from light. Set the illumination light of the DVA system (530 nm) to the max. 5% (equivalent to 30 lux) for positioning.
- Switch the room light off before removing the rat's eye coverage and place the DVA in front of the left eye. Position the camera at an angle <90 degrees and slightly align it from earlier (see Figure 2). *Note: In our experience, this angle allowed the depiction of the papilla, which was necessary for the preinstalled imaging software. The correct positioning and alignment of the camera were essential for the quality of the image and correct measurements*.
- Continuously moisten the rat's left eye and ensure that the pupil is fully dilated before starting the measurement.
- Align a custom-made contact lens (Ocular Instruments, Bellevue, USA) mounted on a micromanipulator centrally in front of the rat's left eye. Then, place the DVA lens so close to the contact lens that a circular cone of light is visible on the contact lens. The DVA lens and the contact lens must be aligned parallel to ensure an identical incidence angle of the light. *Note: Give attention to any Vibrissae or hairs between the eye and the lens and remove them if necessary*.
- Fine-tune the camera settings *via* imaging software to achieve an alignment in which the optic disk is centered. Further optimize the image quality by opening or closing the aperture or adjusting the brightness.

#### 3.5.2. Preparing the measurement

**Figure 4:**
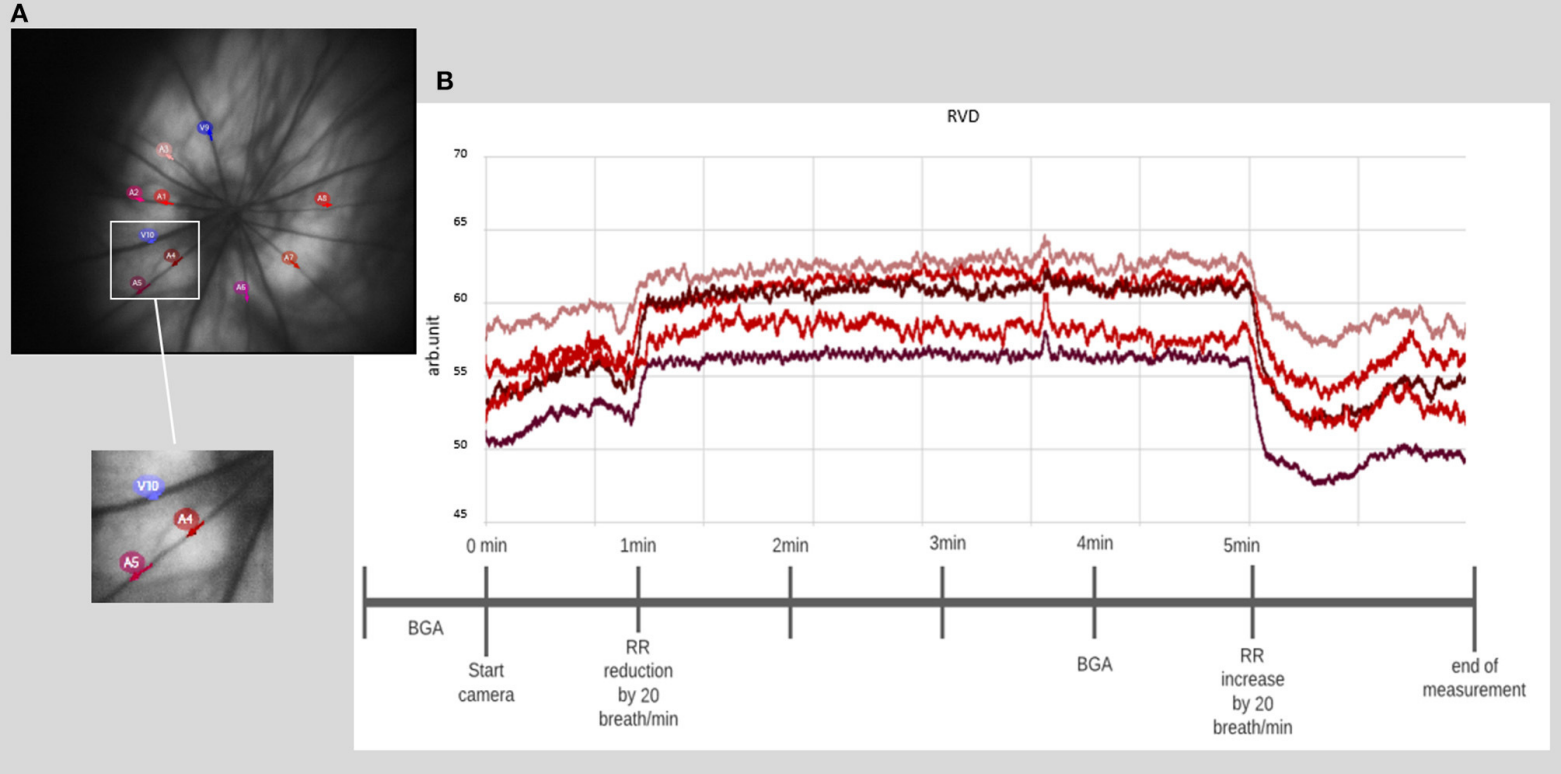
Retinal vessel diameter measurement. **(A)** Example of a fundus image with the selection of 10 different vessel segments. **(B)** Example of a time course of chosen vessel segments, with a prominent increase of the diameter directly after the onset of induced hypercapnia at 1 min, lasting until hypercapnia is ended at 5 min (BGA: blood gas analysis and RR: respiratory rate).

- Perform the image quality check *via* the preinstalled software. If the check is successfully completed, a static image automatically appears to select the corresponding vessel areas. *Note: Deactivation of the built-in image quality check is not recommended to ensure measurement quality*.
- Mark the desired number of vessel sections. *Note: Due to the illumination at 530 nm sensitive to hemoglobin oxygenation, the identification of veins, which appeared darker and wider, and arteries is simple. We recommend a 2.5 times papillary diameter distance for laying the ROIs. It is also important to select vessel sections that have the highest contrast and sharpest image quality (see* *Figure 4**)*.
- Wait for the system to check and confirm the selected areas. In case of rejection, re-marking or selection of a new vessel area must be performed. *Note: By starting the video recording tool, detailed offline analysis is possible. The length of the video recordings and associated analyses can be individually set and adjusted according to the needs of the experimental schedule*.

### 3.6. Protocol of the hypercapnia challenge

In this study protocol, hypercapnia challenges were induced to test and compare the regulatory function of the retinal and cerebral vessel reactions.

- First, perform a blood gas analysis for the baseline value of the arterial partial pressure of carbon dioxide (pCO_2_).
- Immediately afterward, reduce the respiratory rate by 20 breaths per minute, while at the same time increase the oxygen flow to the ventilation system to avoid a drop in the partial pressure of oxygen.
- After 4 min, perform another blood gas analysis to calculate the increase in arterial pCO_2_.
- After 5 min, increase the respiratory rate by 20 breaths per minute back to baseline and reduce the oxygen flow accordingly.

## 4. Results

### 4.1. Cerebral blood flow measurement

The experimental setting described in this protocol ensured stable CBF assessment over a measuring period of up to 6 h. As the bone lamella of the cranial window was thinned to translucency but remains intact, it allowed CBF measurement not only under physiological but also under pathological conditions where an increase in intracranial pressure may occur. The coverslip of the cranial window and the filling with saline ensured stable imaging conditions for the laser speckle contrast analysis. By additional white light illumination, our custom-made laser speckle system also allowed the assessment of changes in hemoglobin oxygenation. However, this measure could not be used in the set-up described herein because the necessary bright light-emitting diodes interfered with the retinal measurements and thus had to be switched off.

### 4.2. Retinal vessel analysis

Our protocol intended multiple replications of retinal assessment. In the described setting, we performed six retinal vessel analysis sessions per animal over a period of up to 6 h. The measuring quality was strongly connected to continuous moistening of the cornea and full dilation of the pupil and a minimum of motion artifacts of the animal or due to bulb rotation.

Positioning the DVA system on a lifting jack allowed three-dimensional movement of the camera for pre-adjustment and stable fixation during the measurement. The preinstalled software tracked the selected vessel sections and could follow them for a small range of movement. However, this function was quickly exhausted. Especially during post-analyses following the actual measurements, movements proved to be particularly disturbing. Sufficiently deep anesthesia and the use of a muscle relaxant prevented movements of the animal itself and bulb rotation, respectively, which frequently occurred despite deep anesthesia.

In general, using as little light as possible during measurements and turning the light off between measurement periods spared the retina and ensured a stable quality in the further course of the measurement. This procedure is particularly important during functional stimulation experiments by flicker light in rodents to prevent light-induced exhaustion of the retinal photoreceptors. It was also essential to maintain constant light conditions during the measurement. The shielding of daylight, room light, and light from devices (e.g., computer screens) was particularly important to improve imaging quality.

### 4.3. Measurement of cerebral reactivity to hypercapnia

**Figure 5:**
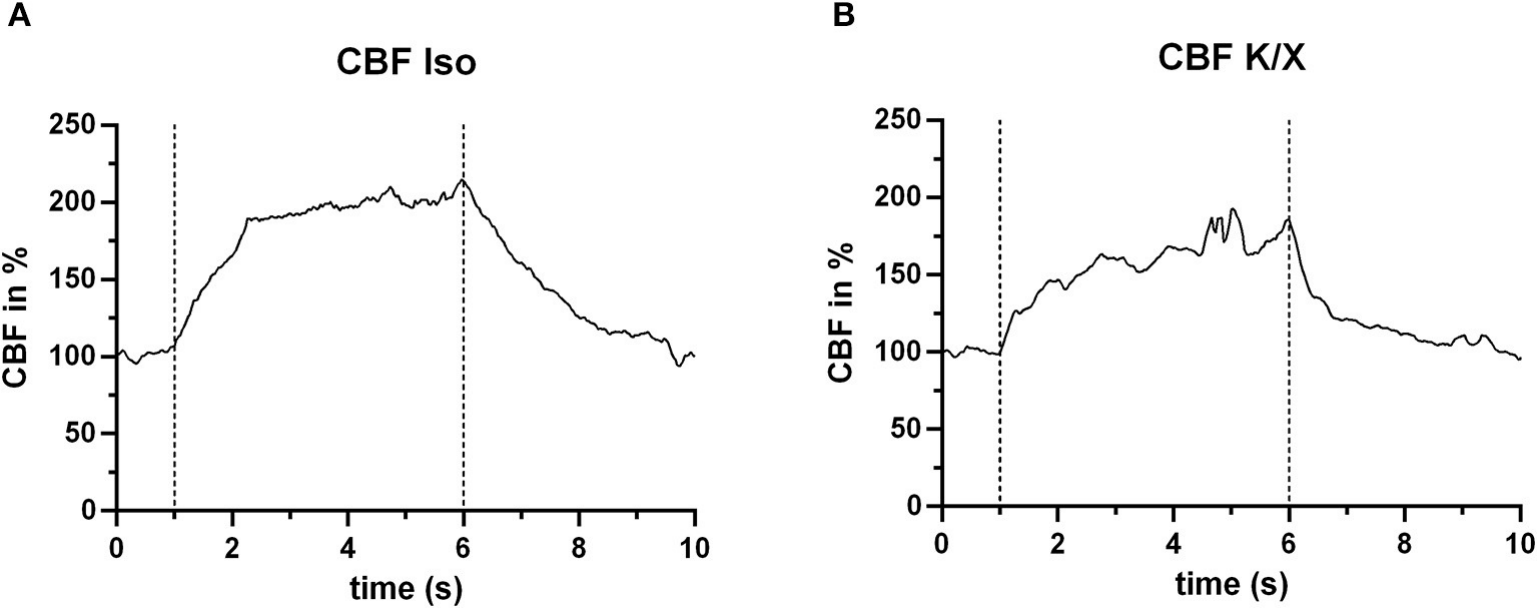
CBF increase during hypercapnia. In both anesthetic protocols, an increase in blood flow in response to hypercapnia (within the dashed lines) was measured. For this example, *n* = 2 animals were used. **(A)** Example under Iso anesthesia (*n* = 1 animal), showing a mean increase in CBF of 84.2% (averaged for the entire hypercapnia phase between the dashed lines), and **(B)** example (*n* = 1 animal) under K/X anesthesia, with a mean CBF increase of 58.0%.

The laser speckle system could reliably measure the blood flow increase during hypercapnia in both anesthetic protocols. For illustration, two typical examples from both groups of anesthesia are given in Figure 5. A detailed analysis of the CBF responses to repetitive hypercapnic stimulation events is provided in our recent publication (see Figure 2 of the Supplementary material in Warner et al., 2022), demonstrating stable reactivity over time with a tendency toward smaller responses in K/X compared to Iso.

### 4.4. Measurement of retinal reactivity to hypercapnia

**Figure 6:**
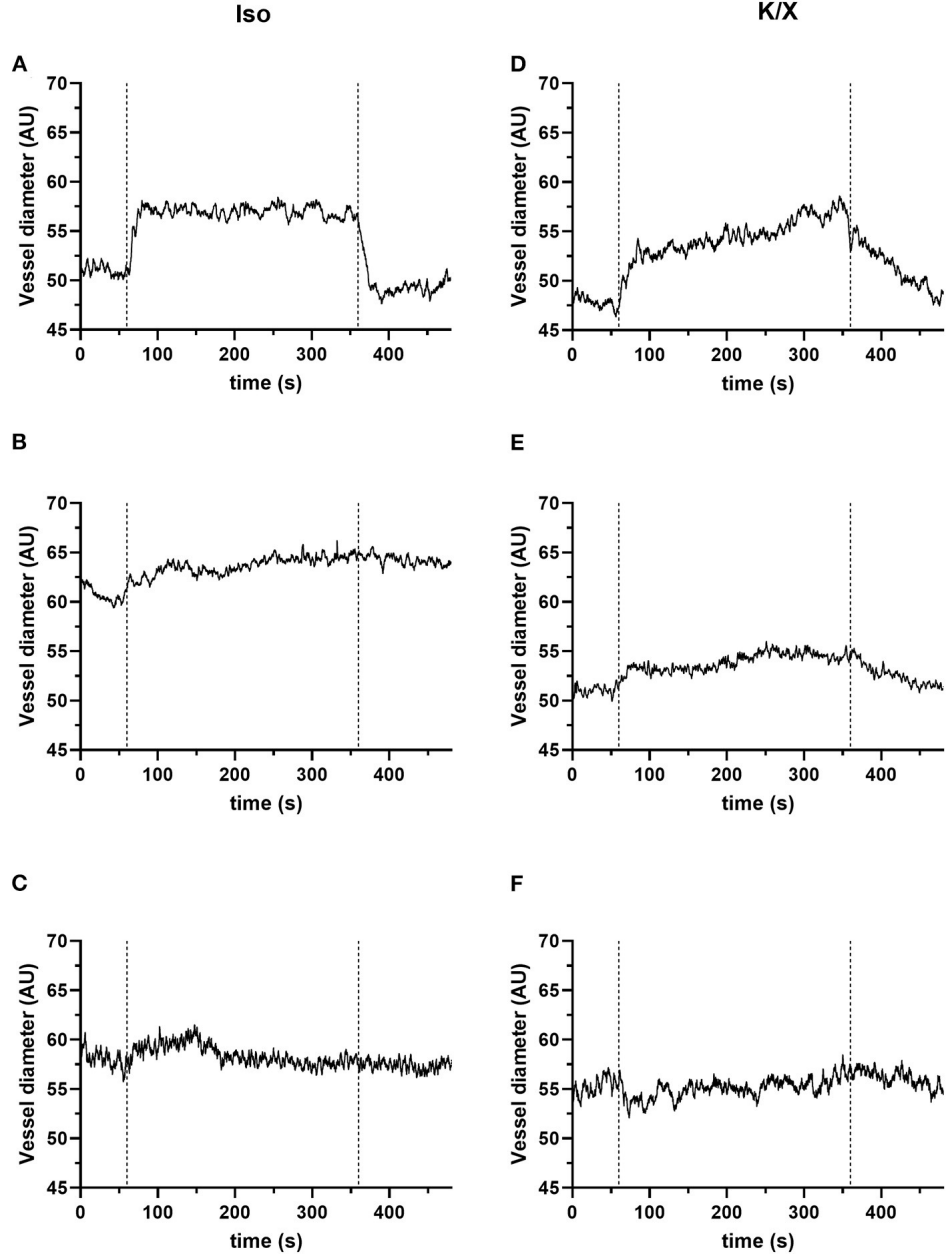
Retinal reactivity to hypercapnia. The retinal reactivity is not consistent in both anesthetic protocols. They can be divided into good **(A, D)**, moderate **(B, E)**, and poor or no **(C, F)** reactivity. For the quantification of the vascular reaction, calculation of mean values of the vessel diameter (in arbitrary units, AU) for a 1-min baseline (immediately before the onset of hypercapnia) and the 5 min during hypercapnia can be performed. For this exemplary illustration, *n* = 3 animals were used for the isoflurane protocol and *n* = 3 animals for the K/X protocol. Category good: **(A, D)**; examples of pronounced vessel diameter increases to hypercapnia: **(A)** in the Iso protocol example, the mean value of the vessel diameter increased from 51.21 to 56.98 AU, corresponding to an increase of 11.28%. **(D)** There were also clear responses in the K/X protocol with a vessel increase of 14.07% (an increase from 47.88 to 54.64 AU). Category moderate: **(B, E)**; examples of moderate responses to hypercapnia; **(B)** in the Iso protocol example, mean vessel diameter increased from 60.97 AU at baseline to 63.74 AU during hypercapnia, representing an increase of 4.6%. **(E)** In the example of the K/X protocol, the vessel diameter increased by 5.64% (baseline 51.06 AU, hypercapnia 53.94 AU). Category poor/no reactivity: **(C, F)**; examples showing no reaction; **(C)** example from the Iso protocol (mean values: baseline 58.11 AU, hypercapnia 58.23 AU, corresponding to an increase of 0.22%). **(F)** Example from the K/X (mean values: baseline 54.99 AU, hypercapnia 55.29 AU, corresponding to an increase of 0.55%).

The measurements of the vessel diameter of the retinal vessels did not give as homogeneous results as those of the cerebral vessels. The detailed analysis provided in our recent publication (see Figure 2 of the Supplementary material in Warner et al., 2022) showed stable reactivity over time with only selecting vessels with good reactivity at the beginning of the measurement phase. Considering all vessel segments at baseline, a higher variability is evident. The individual responses can be roughly divided into good, moderate, and poor/no reactivity. For illustration purposes, exemplary results within the mentioned categories are shown for each anesthetic group in Figure 6.

### 4.5. Essentials for long and/or repetitive measurements

To obtain reliable measurements over a longer period (up to 6 h with six measurements were tested in this study protocol), continuous moistening of the eye, directly at the onset of anesthesia, was essential. For this purpose, transparent ophthalmic gel proved to be the best choice; moistening with the lens in place can also be performed by dropping physiological sodium chloride solution on the eye. If the measurements are preceded by a longer surgical phase as in our study, a coated swab should cover the eye or surgical closure of the lid should be considered. During the measurements, it became apparent that the fitted contact lens significantly improved image quality and also protected the cornea from drying out. To perform retinal measurements, it is necessary to fully dilate the pupil using a mydriatic agent. Alpha antagonists must be avoided, as they can influence the diameters of the retinal vessels and thus falsify the results (Garhofer et al., 2010). We, therefore, used a muscarinic antagonist such as tropicamide. In our experience, poor quality of the retinal images was mostly associated with either dehydration of the cornea or diminishing mydriasis of the pupil.

## 5. Discussion

In this protocol, we propose a method of simultaneous assessment of CBF and retinal vessel analysis. The protocol is proven for generating stable data in CBF assessment over a measuring period of up to 6 h. The retinal set-up is suitable for multiple replications of assessment, but a certain learning curve and more inhomogeneous results must be considered. We give an application example of performing a hypercapnia challenge and outline important pitfalls and troubleshooting. Integrating both measurements in the same animal enables a direct comparison of the two vascular beds in normal and pathological conditions. With the retina as the new hope for easier access to cerebral circulation in a variety of neurovascular and neurodegenerative diseases, the described method could add important aspects to this growing Research Topic. Both fundamental research and clinical application could benefit from supplementing or even replacing this non-invasive assessment in the future. However, during our study, it became apparent that a good familiarization with the retinal camera system set-up and its adjustment options is essential. Accurate image adjustment leads to better, contrasty, and sharp image quality. Reflections, which can occur due to incorrect positioning of the camera and lens, are thus eliminated. Should an exact alignment of the camera fail, it is often advisable to start from the beginning and to readjust and optimize the alignment of the lens and camera. Measurements should not be taken longer than necessary to improve image and measurement quality. Small incidences of light from outside or from equipment degrade the image quality enormously and should be avoided. We, therefore, recommend darkening the experimental room and additionally shielding the animal from artificial lights such as computer screens.

Furthermore, acquiring data from different techniques on the same animal reduces the number of animals needed for experimentation. This follows the current principle of the 3 Rs (Replacement, Reduction, and Refinement) (MacArthur Clark, 2018).

Finally, we present two common anesthetic protocols and show examples of how cerebral and retinal vessels react in baseline and during hypercapnia. In our experience, both protocols proved to be well-controllable and had a sufficient depth of narcotics and analgesia to ensure animal welfare even in a longer acute setting. Thus, we similarly recommend both anesthetic protocols. However, we strongly suggest additional monitoring parameters such as heart rate or blood pressure as pain reflexes are not meaningful anymore after the application of a muscle relaxant. In addition, stable systemic physiology is a prerequisite for normal vascular reactivity. The use of local anesthesia for the eye may also be considered. It is unclear here whether this has an influence on the vascular diameter of the retinal vessels. Due to a sufficient depth of anesthesia and the non-invasive function of the camera system, we refrained from using it.

In retinal research, the pre-experimental adaption of the retina to darkness is often performed (Tan et al., 2017). In our set-up, this adaption was waived as pilot experiments showed no beneficial effects on retinal imaging quality. Due to a comparably long surgical preparation phase before the first retinal assessment, where a minimum of illumination is mandatory, the effects of darkness adaption may be overridden. The vessel reactivity to hypercapnia is a pure vascular response and occurs independent of changes in neuronal activity in the brain as well as in the retina, making dark adaptation unnecessary. Dark adaptation may be relevant for functional stimulation studies in rodents using flicker light to prevent light-induced exhaustion of the retinal photoreceptors. Longer surgeries must then be performed under significantly dimmed light.

As our previous experimental set-up in neurovascular research was tailored to albino Wistar rats, we stick to the model here in the presented protocol. In albino rats, residual optical reflexes of the fundus image rarely occur, but due to a greater penetration depth, more choroidal vessels are visible (Link et al., 2011). Thus, we cannot exclude a certain influence on image quality.

The measurements of retinal vessel diameter have shown that the desired increase in vessel diameter during hypercapnia did not reliably occur in every selected vessel section. This must be taken into account in the study design and calculation of animal numbers. Further systematic analyses are needed to find reasons for the lack of response of some vessel sections.

### 5.1. Applications of the design

This method is not limited to the acute set-up described here. The retinal assessment is non-invasive and, therefore, can be repeated as often as desired, with a rest of at least 30 min between measurements recommended for studies in humans which may also account for rodents.

By the modification of the surgical procedures, the simultaneous assessment of cerebral and retinal vessels can also be performed in chronic set-ups to study long-term alterations of both vessel beds. Since a muscle relaxant is used, artificial ventilation is mandatory. This can be performed orotracheally and thus non-invasively for a possible chronic study. Vecuronium, as the muscle relaxant used, has a short half-life, so it is possible to wean animals off ventilation after a short anesthetic.

### 5.2. Limitations

The described protocol offers the possibility to measure the cerebral and retinal vascular beds simultaneously, but it must be noted that the systems presented capture different parameters. The laser speckle system measures blood flow in a defined region of interest within the microcirculation, whereas the DVA can measure the vessel diameter of rather larger retinal vessels. A correlation of these parameters is assumed but still needs to be verified experimentally.

### 5.3. Conclusion

We demonstrate the utility of simultaneous cerebral blood flow and retinal vessel assessment in rats using a laser speckle system *via* a closed brain window and a non-invasive retinal vessel analyzer, with some detailed method specifications related to the Imedos-RVA system. The design is applicable to multiple acute and—with slight modifications in surgical preparation—chronic settings. Thus, the effects of acute or subacute neurovascular events such as hemorrhage, stroke, or trauma can be observed and monitored in real-time. This allows a direct comparison of both vascular beds and substantiates the role of the retina as a “window to the brain.”

## Data availability statement

The original contributions presented in the study are included in the article/Supplementary material, further inquiries can be directed to the corresponding author.

## Ethics statement

The animal study was reviewed and approved by Landesamt für Natur, Umwelt und Verbraucherschutz (LANUV), Recklinghausen, Germany.

## Author contributions

CC-D, GS, and UL: conceived and designed the experiments and the study protocol. LW, AB-H, WA, SP, UL, and CC-D: constructed experimental set up. LW: performed the experiments. LW, CC-D, and UL: interpretation of the data and illustrations. CC-D and LW: first drafting of the manuscript. AB-H, TS, GS, WA, UL, HC, SP, and CC-D: critical review of the manuscript. All authorship requirements have been met and the final manuscript was critically revised and approved by all authors.

## Supplementary material

Supplementary Video 1 Recording of a rotating eyeball when a muscle relaxant is not used.
